# Association between rs2188380 and the risk of breast cancer in southwest Chinese population

**DOI:** 10.1002/jcla.22889

**Published:** 2019-03-29

**Authors:** Lin Huang, Jinling Liao, Yang Chen, Zengnan Mo

**Affiliations:** ^1^ Department of Urology The First Affiliated Hospital of Guangxi Medical University Nanning China; ^2^ Institute of Urology and Nephrology The First Affiliated Hospital of Guangxi Medical University Nanning China

**Keywords:** association, breast cancer, gout, rs2188380

## Abstract

**Background:**

Previous studies have revealed that the single nucleotide polymorphism (SNP) rs2188380 was identified as a novel locus of gout. Interestingly, gout resulting from high serum uric acid (SUA) was also identified to be associated with the risk of breast cancer (BC). We hypothesized that maybe there was a relationship between rs2188380 and the risk of BC. Therefore, our study was conducted to investigate whether this novel gout‐related SNP (rs2188380) was associated with BC risk as well as the clinical and pathological characteristics in the southwest Chinese population.

**Materials and methods:**

We performed a case‐control study including 104 breast cancer patients and 112 healthy controls to investigate whether rs2188380 is associated with BC risk in the southwest Chinese population. The genotyping was performed by the SNP scan method. General characteristics and clinicopathological characteristics of tumors were also included in the analysis. The statistical evaluations were performed using the Student t test, the chi‐square test or Fisher's exact test, and unconditional logistic regression analysis.

**Results:**

The C/C genotype of rs2188380 might be related to BC risk to some extent compared with G/G genotype (OR = 9.241, 95% CI = 1.122‐76.101, *P *= 0.039). Furthermore, after adjusting the age, the association still existed (OR = 8.788, 95% CI = 1.063‐72.636, *P* = 0.044). However, no significant association was observed between rs2188380 and the clinicopathological characteristics of BC.

**Conclusions:**

Our study primarily indicated that rs2188380 might have a potential association with BC risk to some extent. With a limited sample size and statistical power, further studies based on larger populations are needed to confirm the association.

## 1 INTRODUCTION

Breast cancer (BC) is one of the most common malignant tumors in women accounting for almost one in four cancer cases among women and with about 2.1 million newly diagnosed cases in 2018.^1^ Genetic factors may play an important role in the BC development.^2^ Till now, a larger number of genetic susceptibility variants and loci have been discovered.^3^, ^4^

rs2188380 is located on an intergenic region between *MYL2* and *CUX2* genes of chromosome 12 (genes associated with cholesterol^5^ and diabetes mellitus^6^). MYL2 encodes a regulatory light chain associated with cardiac myosin β heavy chain and is associated with high‐density lipoprotein cholesterol metabolism.^5^ CUX2 regulates cell‐cycle progression, and its association with type 1 diabetes has also been reported.^6^ Recently, a genome‐wide association study (GWAS) in Japan was conducted with 945 gout patients and 1213 controls in stage 1, and 1048 cases and 1334 controls in stage 2, which suggested that rs2188380 was a novel gout‐related locus.^7^ Additionally, another study^8^ with 1048 gout patients and 1334 controls also revealed that rs2188380 might play a role in the development of gout.

Gout is a common disease resulting from high serum uric acid (SUA). Interestingly, high SUA (>5.41 mg/dL) was independently associated with increased risk of total cancer mortality and further positively related to deaths from malignant neoplasms of breast.^9^ Meanwhile, the serum uric acid concentration was positively correlated with the advanced stages (TNM stages IIIc and IV) in breast cancer compared with early stages (TNM stages I and II).^10^ Moreover, high SUA concentration predicted a poor survival in breast cancer patients.^11^ Therefore, we hypothesized that there may be a relationship between gout‐related single nucleotide polymorphisms (SNPs; rs2188380) and BC risk. In order to verify this hypothesis, our study was conducted to assess the association between rs2188380 and BC risk as well as the clinicopathological characteristics comprehensively among Guangxi population in the southwest China.

## 2 MATERIALS AND METHODS

### 2.1 Ethics statement

The study had been approved by the Institutional Ethics Review Board of the Guangxi Medical University. The informed consent was obtained from all participants.

### 2.2 Subjects

We conducted a case‐control study that included a total of 104 female BC patients evaluated by immunohistochemical staining and 118 healthy controls from 2013 to 2015. All subjects were recruited from the First Affiliated Hospital of Guangxi Medical University, Nanning City, the People's Republic of China (PR China). All the participants had no history of cancer or hereditary diseases in their families and had no blood relationship. The patients and healthy controls were independent without familiarity. The controls were without previous personal and familial cancer history and diagnosed psychiatric diseases. Meanwhile, they had no long‐term use of medicines (such as glucocorticoids). All cases were pathologically confirmed by immunohistochemical staining without preoperative chemotherapy or radiotherapy. The clinical and pathological information such as age, body mass index (BMI), race, menarche age, menopause status, metastasis, molecular classification, and pathology type was obtained from medical files. The stratified classification of these clinicopathological characteristics was described in our previous study^12^: BMI: non‐obese <23.9 kg/m^2^ and obese ≥24 kg/m^2^, and race: Han and other groups. The metastatic status was according to the tumor‐node‐metastasis (TNM) classification. The molecular type was performed by the 2011 St. Gallen Consensus.^13^

### 2.3 DNA extraction and genotyping

The blood samples were frozen immediately after collection and stored at −80℃ until use. The genomic DNA was extracted from whole blood samples from cases and controls with a DNA isolation kit (Sangon Biotech, Shanghai, China). The genotyping of rs2188380 was performed by SNPscan system (Genesky Biotechnologies Inc, Shanghai, China). SNPscan technology is a propriety multiplex SNP genotyping system technology that uses the specificity of the ligase‐linked reaction to identify the SNP allele. Details of the technology were described in the study (http://biotech.geneskies.com/en/index.php/Index/fuwuer/id/29). To evaluate the quality of genotyping, 10% samples from cases and controls were selected randomly for replication testing and no discrepancies were found.

### 2.4 Statistical analysis

The results of clinical parameters were shown as mean ± standard deviation or number of cases (%). Qualitative data were analyzed by chi‐square test or Fisher's exact, and quantitative data were analyzed by Student's *t* test. The chi‐square test or Fisher's exact test was performed to analyze the association between the rs2188380 and various clinicopathological features of BC. Simple unconditional logistic regression and multiple logistic regression analyses with an odds ratio (OR) and 95% confidence interval (CI) were applied to assess the association between BC risk and genotype distribution or population characteristics. Statistical data in the whole analysis were analyzed by IBM® SPSS® Statistics version 22.0. All statistical tests were two‐sided, and *P* values <0.05 were considered statistically significant.

## 3 RESULT

### 3.1 Subject characteristics

The baseline characteristics of 104 patients and 118 healthy controls are summarized in Table 1. Overall, the general characteristics of cases and controls in this study were matched adequately as the distribution of age, BMI, race, and age at menarche between the two groups were of no significant difference.

**Table 1:** The characteristics of the study population

Variable	Case (n = 104)	Control (n = 118)	*P‐*value
Age (y)	47.8 ± 10.1	46.4 ± 10.9	0.832^a^
BMI (kg/m^2^)	23.0 ± 2.9	22.7 ± 3.1	0.746^a^
Menarche age (y)	14.1 ± 1.7	14.6 ± 1.9	0.115^a^
Race			
Han	80	89	0.794^b^
Others	24	29	
Menopausal status			
Premenopausal	49		
Postmenopausal	54		
Metastasis			
No	92		
Yes	12		
Molecular classification			
Luminal A	5		
Other	99		
Luminal B1	49		
Other	55		
Luminal B2	24		
Other	80		
Her‐2 overexpression	6		
Other	88		
Triple‐negative	9		
Other	95		
Pathology type			
Ductal	86		
Lobular	3		
Other	15		

BMI, body mass index.

a Student's *t* test was applied to assessing the difference between cases and controls.

b The chi‐square test.

### 3.2 The polymorphism rs2188380 may have a potential association with the risk of BC to some extent

The genotype frequencies and allelic distribution of rs2188380 polymorphisms are given in Table 2. In cases, the T/T, T/C, and C/C genotypes of rs2188380 locus were 55.8%, 36.5%, and 7.7%, and the corresponding frequencies were 59.8%, 39.3%, and 0.9% in the control group. We found that the C/C genotypes (OR = 9.241, 95% CI = 1.122‐76.101, *P* = 0.039) of rs2188380 had an increased BC risk compared with G/G genotype. When age as a confounding factor was adjusted, the C/C genotypes of rs2188380 still had an increased BC risk (OR = 8.788, 95% CI = 1.063‐72.636, *P* = 0.044). In patients' group, the frequency of T and C was 74% and 26%. The frequency of T and C was 79.5% and 20.5% in control group, and no significant differences were observed in the frequency distribution of per‐allele between two groups (*P* = 0.148).

**Table 2:** The genotype distribution of rs2188380 polymorphisms in cases and controls

Genotype	Case (n = 104)		Control (n = 112)		OR (95% CI)	*P*‐value	OR (95% CI)*	*P*‐value*
	n	%	n	%				
rs2188380								
T/T	58		67		1.000	–	1.000	–
T/C	38		44		0.998 (0.571‐1.744)	0.993	0.993 (0.567‐1.737)	0.979
C/C	8		1		9.241 (1.122‐76.101)	0.039*	8.788 (1.063‐72.636	0.044**
Allele								
T	154		178		1.000	–	–	–
C	54		46		1.395(0.889‐2.188)	0.148	–	–

CI, confidence interval; OR, odds ratio; SNP, single nucleotide polymorphism.

Statistical test performed by simple unconditional logistic regression. rs12922061 T/T genotype was considered as a reference.

* Age as a confounding factor was adjusted.

** Statistically significant.

The association between rs2188380 genotype distribution and clinical characteristics in patient group is shown in Table 3. However, no significant association was observed between rs2188380 and clinical characteristics including age, BMI, race, menarche age, and menopausal status in the patient group. As shown in Table 4, no significant associations were observed between rs2188380 and clinicopathological characteristics including metastasis, molecular classification, and pathology type in the patient group.

**Table 3:** The relationship between rs2188380 polymorphism and general characteristics

Characteristic	C/C + T/C (n = 46)	T/T (n = 58)	OR (95% CI)	*P*‐value
Age				
<50	23	35	1.000	–
≥50	23	23	1.522 (0.696‐3.325)	0.292
BMI				
<23.9	25	42	1.000	–
>24	21	16	2.205 (0.974‐4.993)	0.058
Race				
Han	37	43	1.000	–
Others	9	15	0.697 (0.273‐1.778)	0.450
Menarche age				
≤14	33	40	1.000	–
>14	13	18	0.875 (0.374‐2.047)	0.759
Menopausal status				
No	19	30	1.000	–
Yes	26	28	1.466 (0.669‐3.212)	0.339

BMI, body mass index; CI, confidence interval; OR, odds ratio; SNP, single nucleotide polymorphism.

**Table 4:** The relationship between rs2188380 polymorphism and tumor clinic characteristics

Characteristic	Status	C/C + T/C (n = 46)	T/T(n = 58)	*P*‐value*
Metastasis	No	40	52	0.669
	Yes	6	6	
Molecular classification	Luminal A	4	1	0.168**
	Other	42	57	
	Luminal B1	17	32	0.065
	Other	29	26	
	Luminal B2	10	14	0.773
	Other	36	44	
	Her‐2 overexpression	0	6	0.079**
	Other	36	52	
	Triple‐negative	5	4	0.504**
	Other	41	54	
Pathology type	Ductal	36	50	0.113**
	Lobular	3	0	
	Other	8	7	

* Statistical test performed by the chi‐square test (*χ* ^2^).

** Fisher's exact test.

## 4 DISCUSSION

The etiological mechanism of BC has not been fully understood. Nowadays, it is believed that BC is a genetical heterogeneous disease with complex genetic patterns^14^ and the mutation of genes is an important factor in the occurrence and development of breast cancer.^15^ In this study, we aimed to evaluate the association between a novel locus of gout (rs2188380 of MYL2‐CUX2) and BC risk as well as the clinical and pathological characteristics in a total of 104 BC cases and 112 control individuals. We found that the rs2188380 may have a potential association with BC risk to some extent.

Single nucleotide polymorphisms are the most frequent variation that occur in a single nucleotide at a specific position in the genome. Previous studies showed that some SNPs in critical genes were identified to be associated with BC susceptibility. Tian et al^16^ found that the CA/AA, CA genotype, and the A allele of rs3761548 could increase the risk of BC in a Chinese Han population. Moreover, the A allele of rs3761548 was associated with a larger size of tumor and more likely to have overexpression of *HER2*, which means that this locus might help to guide treatment for BC and be a potential biomarker for classification of tumor subtype. In another Chinese population, Di et al^17^ reported that rs28366003 and rs10636 polymorphisms in MT2A had a correlation with BC risk; moreover, the relationship between this polymorphism and the histological grade might help us to judge tumor prognosis.

Recently, a GWAS^3^ in a Japanese population showed that the rs2188380 of MYL2‐CUX2 was identified as a novel gout locus. Furthermore, another study^8^ revealed that the SNP rs671 around rs2188380 of *MYL2‐CUX2* was also a genuine gout‐associated SNP. Gout is a common disease resulting from hyperuricemia. Hyperuricemia is a marker of oxidative stress. As the pro‐oxidant properties and other abilities including the inhibition of lipid peroxidation, impediment of inflammation, and scavenge singlet oxygen, increased uric acid levels may lead to hyperuricemia‐related tumorigenesis, which implicates an underlying link between purine metabolism disorders and cancer.^18^

As for breast cancer, Strasak et al^9^ conducted a large cohort study in 28613 Austrian gout women, and after a median 15.2 years of follow‐up, the results showed that higher SUA was independently correlated with an increased risk of the cancer mortality of breast cancer. Patients with the highest quartile of uric acid increased their overall risk of cancer death by 27% compared with the lowest quartile group. Yue et al^11^ performed a study that included 443 female breast cancer patients to investigate the association between SUA and the risk of breast cancer, and after a 56 months of follow‐up, a poor OS was observed in patients with relative higher SUA concentration, which confirmed a significant association of increased uric acid concentration with poor clinical outcome in breast cancer patients. Another study conducted by Panis et al^10^ showed that SUA concentration in advanced breast cancer (TNM stages IIIc and IV) was significantly higher than that in early stages of breast cancer patients (TNM stages I and II) and control group. The SUA concentration was positively correlated with advanced stages in breast cancer, which suggested that the increased SUA might be used as an index for development of breast cancer. The results also indicated that oxidative stress enhancement and immune response impairment that resulted from tumor inflammatory mediators may play an important role in breast cancer progression.

As rs2188380 was identified as novel locus of gout and high SUA was associated with risk of breast cancer, we hypothesized that there may be a relationship between rs2188380 and risk of breast cancer. To some extent, our study indicated a potential association. In our study, the C/C genotypes of rs2188380 had an increased breast cancer risk, and after age as a confounding factor was adjusted, the result was also positive. Additionally, we thought that rs2188380 might influence breast cancer through some metabolic pathways. Previous studies had shown that rs2188380 of MYL2‐CUX2 was identified as a novel gout locus and MYL2‐CUX2 were associated with cholesterol and diabetes mellitus. Moreover, gout, hypertension, obesity, diabetes, and hyperlipemia are parts of the metabolic syndrome and present a series of conditions associated with chronic low‐grade inflammation and immunological function.^19^ SUA is a marker of oxidative stress linked closely with inflammation^19^ and is supposed to be involved in the pathogenesis of cancer through the effect of pro‐inflammatory cytokines.^20^ In the breast cancer microenvironment, uric acid is regarded as a mediating factor to activate the chemotaxis of mesenchymal stromal cells (MSC) and promote the MSC‐associated immunoregulation.^21^ Uric acid crystals can be recognized as a danger signal by Toll‐like receptor four and then activate leukocytes to produce pro‐inflammatory cytokines, which are associated with many inflammatory diseases. 22 Free uric acid can activate mitogen‐activated protein kinase (p38 and extracellular signal‐regulated kinase) and transcription factor NF‐kb, which induces the expression of inflammatory mediators including monocyte chemoattractant protein‐1 and C‐reactive protein.^23^ These effects may be mediated by NADPH oxidase‐induced oxidative stress.^24^ On the other hand, uric acid can activate the dendritic cell to mature and strengthen the responses from CD8+ T cells, which have been regarded as a mechanism through the immune system in response to tumors.^25^ Additionally, factors such as adiponectin,^26^ leptin,^27^ and C‐reactive protein^28^ are also involved in uric acid‐related inflammation that is associated with cancer risk.

This study has some limitations worthy of note. Firstly, restricted by the conditions, the number of related patients and controls in this study is relatively small. Especially in subgroups, statistical power may be limited to finding differences between the groups. Secondly, our research cases and controls were selected from only one hospital; therefore, the selection bias could not be avoided. Further studies with larger sample size in multi‐center population are needed. Thirdly, although our results indicated that rs2188380 between MYL2 and CUX2 may be related to BC risk to some extent, the 95% CI is relatively wide. We think the primary reason may be the number of related research cases, especially subgroups, which is small as restricted by the conditions. Additionally, relatively small cases may be the same reason for no statistical differences between rs2188380 genotype and clinicopathological characteristics.

## 5 CONCLUSIONS

Our study primarily indicated that rs2188380 might have a potential association with breast cancer risk to some extent, and it may affect breast cancer through some metabolic pathways. However, no significant association was observed between rs2188380 genotype and clinicopathological characteristics of breast cancer. Further studies with larger sample size and multi‐center population are necessary to confirm the association.
